# Mathematical Competencies and Critical Thinking in Secondary Education: A PRISMA-Based Systematic Review (2019–2025)

**DOI:** 10.12688/f1000research.173462.2

**Published:** 2026-01-21

**Authors:** Nevy Alvarez-Tinajero, Andrea Basantes-Andrade, Orlando Ayala-Vásquez, Luz-M Pereira-González, Gabriela Arciniegas-Romero

**Affiliations:** 1 Grupo de Investigación, Educación, Ciencia y Tecnología GIECYT, Universidad Tecnica del Norte, Ibarra, Imbabura Province, 100105, Ecuador; 2 Network Science Research Group (eCIER), Universidad Tecnica del Norte, Ibarra, Imbabura Province, 100105, Ecuador

**Keywords:** Critical thinking, mathematical competencies, secondary education, systematic review, PRISMA 2020, active methodologies, STEM, problem-based learning

## Abstract

**Background:**

The integration of mathematical competencies and critical thinking in secondary education has become increasingly relevant to equip students with the ability to reason, argue, and solve complex real-world problems. Despite its recognized importance, this integration remains fragmented across curricula and inconsistently applied in classroom practice.

**Methods:**

A systematic review was conducted in accordance with PRISMA 2020 guidelines to analyze empirical evidence published between 2019 and 2025. Searches were performed in Scopus, Web of Science, SciELO, and Dialnet databases. Inclusion criteria focused on open-access, peer-reviewed studies in English or Spanish addressing the integration of mathematical competencies and critical thinking in secondary education. From 1,457 records initially retrieved, 24 empirical studies were selected for full analysis.

**Results:**

The studies reviewed indicate that mathematical competencies, defined as the ability to model, interpret, and solve problems through logical reasoning, are reinforced when integrated with critical thinking skills such as evaluation, argumentation, and evidence-based decision-making. Active methodologies, including Problem-Based Learning (PBL), Project-Based Learning (PjBL), STEM, and gamification, were identified as the most effective strategies to foster this integration. However, their implementation is often constrained by structural barriers such as limited teacher training, rigid curricula, and insufficient technological infrastructure.

**Conclusion:**

Integrative, student-centered approaches supported by active learning methodologies enhance higher-order cognitive development and prepare learners to meet the demands of 21st-century education. Future research should address methodological standardization, teacher preparation, and institutional conditions to ensure the sustainable and equitable implementation of these strategies.

## 1. Introduction

In the face of 21st-century challenges such as digital transformation, labor market uncertainty, and increasing social complexity, secondary education is confronted with the pressing need to redefine the competencies students must develop. In this context, competence development must move beyond mere knowledge acquisition and focus instead on the ability to mobilize cognitive, emotional, and social resources to act critically, autonomously, and contextually ( Zhu et al., 2021). Among these, mathematical competence and critical thinking stand out as essential capacities that strengthen academic performance, civic preparedness, and the ability to solve real-world problems ( Pramasdyahsari et al., 2023; Pratiwi et al., 2025; Pereira-González et al., 2024).

This systematic review focuses on secondary education because this stage represents a critical period for the consolidation of abstract, formal, and hypothetical-deductive reasoning, which are foundational for both mathematical competence and critical thinking development ( Inhelder & Piaget, 2013; OECD, 2019). International curricular frameworks emphasize secondary education as the stage where higher-order thinking, problem solving, and analytical reasoning are explicitly articulated as learning outcomes ( UNESCO, 2021; OECD, 2022). Furthermore, secondary school mathematics involves increased conceptual complexity, formalization, and transfer demands, making it a particularly relevant context to examine the integration of mathematical competence and critical thinking.

According to the PISA framework, mathematical competence involves the formulation, use, and interpretation of mathematics across a variety of contexts, highlighting its functional and social dimensions ( Beccuti, 2024). This competence goes beyond numerical operations; it encompasses complex cognitive skills such as logical reasoning, modeling, data interpretation, and evidence-based argumentation. Numerous studies have directly linked mathematical competence to academic achievement, problem-solving ability, and readiness to face novel situations ( Alegre et al., 2019; Wahono et al., 2020; Pramasdyahsari et al., 2023; Pratiwi et al., 2025).

Critical thinking, on the other hand, is defined as the ability to analyze, evaluate, and construct arguments in a reasoned, reflective, and context-sensitive manner ( Rahmatika et al., 2024). Within the educational sphere, it enables students to question assumptions, make informed decisions, and transfer knowledge across diverse contexts. Although multiple theoretical approaches exist regarding its development, authors such as Pereira-González et al. (2024) agree on its transversal and situated nature, meaning it is closely tied to specific domains of knowledge such as mathematics.

The relationship between mathematical competence and critical thinking is not unidirectional but reciprocal and recursive. Mathematical reasoning provides structured contexts for analysis, argumentation, and abstraction, while critical thinking enhances conceptual understanding, problem interpretation, and metacognitive regulation in mathematics ( OECD, 2019; Pratiwi et al., 2025; Pereira-González et al., 2024; Rahmatika et al., 2024). Recent empirical studies confirm that students who engage in mathematical tasks requiring justification, modeling, and decision-making simultaneously develop higher levels of critical evaluation, cognitive flexibility, and reflective thinking ( Li & Oon, 2024; Er, 2024; Pramasdyahsari et al., 2023). This interdependence positions both constructs as mutually reinforcing rather than hierarchically ordered, suggesting that their integration is pedagogically synergistic rather than sequential.

However, the intersection between mathematical competence and critical thinking remains insufficiently explored from an integrative perspective. Despite their natural complementarity, both requiring logical reasoning, interpretation, and contextual analysis, they are often addressed separately in many curricula. This theoretical and practical disconnection is reflected in classrooms, where students struggle to apply mathematical concepts in real-life situations requiring critical judgment ( Er, 2024; Li & Oon, 2024).

Recent literature highlights persistent barriers to the integration of these competencies in secondary education: rigid curricula, lack of teacher training in active methodologies, weak student research culture, and limited use of interactive technologies ( Chacón-Cueva et al., 2023; Cruz et al., 2022; McLure et al., 2022). These limitations hinder pedagogical innovation and compromise the quality of learning during a crucial stage in adolescent cognitive development.

In response to this context, several educational paradigms, such as constructivism ( Vygotsky, 1978), critical pedagogy ( Freire, 1970), and competency-based learning, converge in emphasizing the need to provide meaningful, collaborative, and contextually relevant experiences. Models such as Problem-Based Learning (PBL), Project-Based Learning (PjBL), and STEM education have proven effective in simultaneously fostering critical thinking and mathematical competence ( Mayorga-Aguirre et al., 2024; Setyawati et al., 2022).

Nevertheless, despite increasing interest in this field, there remains fragmentation in the available empirical evidence. Few studies jointly examine both competencies, and even fewer systematically analyze the methodologies employed, their outcomes, and limitations. Therefore, this systematic review aims to critically examine the recent scientific literature on the integration of mathematical competence and critical thinking in secondary education, considering the impact of pedagogical strategies, identified barriers, and emerging trends.

The research questions guiding this review are:

RQ1: How are mathematical competencies and critical thinking integrated in secondary education?

RQ2: What is the impact of integrating mathematical competencies and critical thinking on academic performance and the development of cognitive skills in secondary school students?

RQ3: What are the main barriers and challenges to the integration of critical thinking in mathematics instruction at the secondary level?

## 2. Methods

This systematic review was conducted in accordance with the PRISMA 2020 protocol guidelines ( Page et al., 2021) with the aim of identifying, evaluating, and synthesizing empirical evidence on the integration of mathematical competencies and critical thinking in secondary education.

### 2.1 Information sources and search period

The selection of these sources was based on the objective of capturing scientific literature in both English and Spanish, particularly within the Ibero-American context, without compromising methodological quality. Although databases such as ERIC or ProQuest also offer relevant resources, priority was given to repositories with higher impact factors and editorial control. This selection criterion is acknowledged as one of the study’s limitations, and its expansion is proposed for future research. The search period spanned from January 2019 to March 2025, with the aim of including the most recent publications on the topic. The last search in each database was performed on April 14, 2025, ensuring the currency of the retrieved records.

The time frame 2019–2025 was selected to capture the most recent and relevant evidence following the global curricular shift toward competency-based education and critical thinking development ( OECD, 2019; UNESCO, 2021). This period also reflects the acceleration of digital transformation and active methodologies in education, particularly in response to the COVID-19 pandemic, which significantly reshaped pedagogical practices worldwide.

### 2.2 Inclusion and exclusion criteria

To ensure the relevance of the selected studies, the inclusion and exclusion criteria presented in Table 1 were established.

**Table 1.** Inclusion and exclusion criteria.

Criteria	Inclusion criteria	Exclusion criteria
Type of articles	Empirical articles/Systematic reviews	Non-scientific articles, book chapters, theses, and editorials
Content	Studies related to mathematical competencies and critical thinking	Studies not related to mathematical competencies and critical thinking
Language	Spanish and English	Other than Spanish and English
Type of participants	Secondary school students	Primary school or higher education students

### 2.3 Search strategy

Standardized descriptors from the UNESCO and IEEE thesauri were used to construct the search strings, prioritizing relevant English-language terms to ensure conceptual coverage of mathematical competencies and critical thinking at the secondary education level. The main search string applied was:

(“critical thinking”) AND (“secondary education” OR “secondary school students”) AND (“mathematics” OR “mathematical competence” OR “skills”).

This string was implemented across all selected databases (Scopus, Web of Science, SciELO, and Dialnet), with syntax adjustments made according to the specific requirements of each search engine. The strategy was designed to maximize sensitivity without compromising relevance, enabling the identification of literature related to both critical thinking and mathematical skills in secondary educational settings.

### 2.4 Study selection process

**Figure 1.**
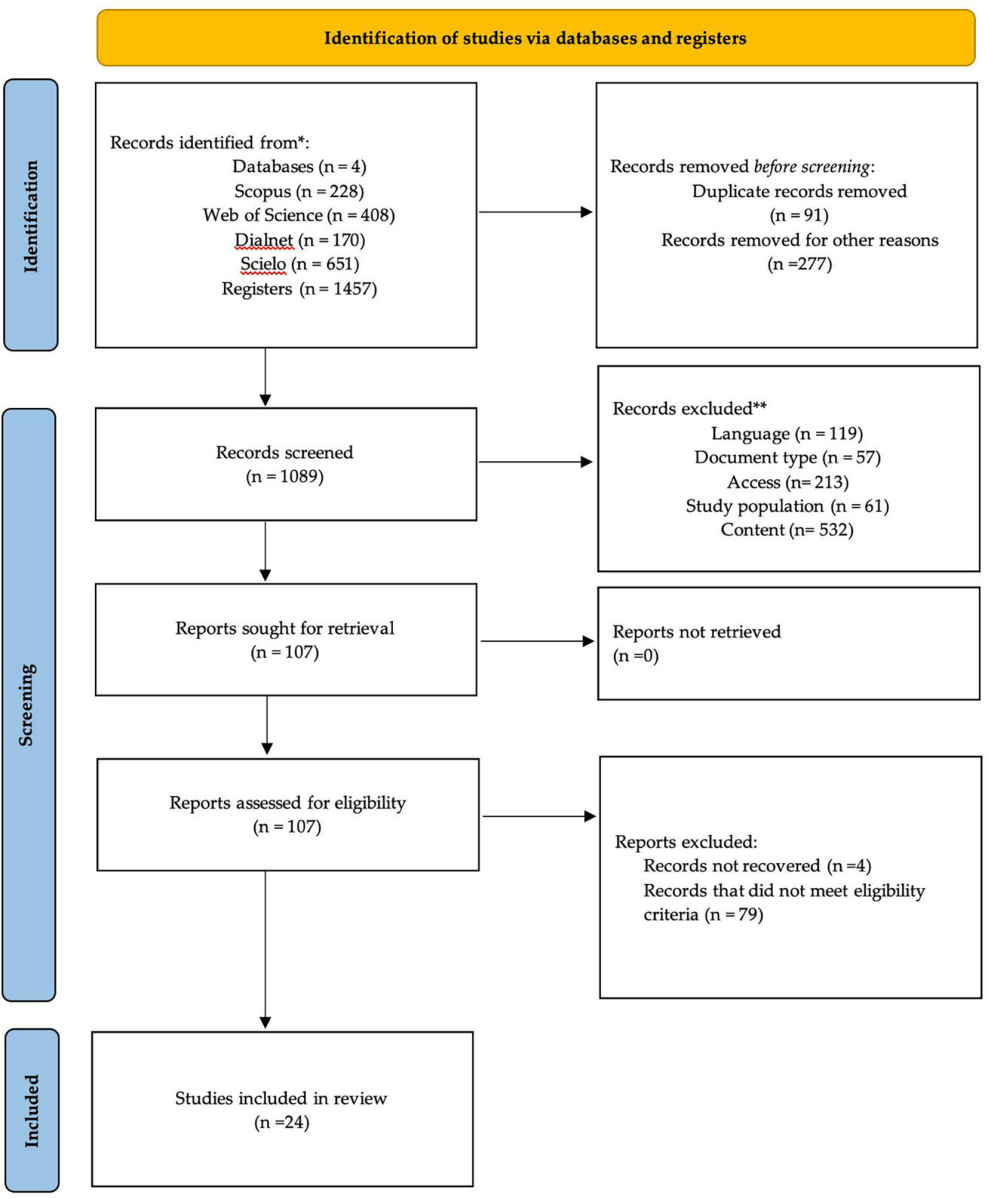
Flow diagram of the systematic review according to PRISMA guidelines.

The identification, screening, and inclusion process is illustrated in Figure 1, following the PRISMA 2020 flow diagram. A total of 1,457 records were initially identified across four databases: Scopus (n = 228), Web of Science (n = 408), Dialnet (n = 170), and SciELO (n = 651). All records were exported to Microsoft Excel, where duplicates (n = 91) and records outside the 2019–2024 publication window (n = 277) were removed, resulting in 1,089 studies for screening. During this phase, records were excluded due to language (n = 119), document type (n = 57), lack of access to full text (n = 213), non-relevant study population (n = 61), and misalignment with the topic (n = 532). This resulted in 107 full-text articles assessed for eligibility. Of these, four could not be retrieved and 79 did not meet the inclusion criteria. Finally, 24 studies were included in the qualitative synthesis. The review was conducted independently by two evaluators. Inter-rater agreement was calculated using Cohen’s kappa (κ = 0.83), indicating a high level of consistency. Discrepancies were resolved through discussion and consensus, which strengthened transparency and minimized selection bias.

The methodological quality assessment was conducted using an ad hoc matrix consisting of ten items, adapted from Khan et al. (2022) and Petticrew and Roberts (2008), designed to evaluate key aspects such as methodological clarity, internal consistency, and theoretical alignment (see Table 2). This matrix was specifically adjusted to accommodate quantitative, qualitative, and mixed-method studies, in line with the exploratory and educational focus of this review. The results of this evaluation are available in open access on Figshare ( https://doi.org/10.6084/m9.figshare.29635733.v1) in the Excel file entitled “Calidad de estudios” ( Alvarez-Tinajero et al., 2025).

**Table 2.** Evaluation criteria.

No.	Question	Criterion
P1	Are the study objectives aligned with the development of mathematical competencies and critical thinking in secondary education?	Yes/Partial/No
P2	Is the methodology used clear and understandable?	Yes/Partial/No
P3	Does the study population include secondary school students?	Yes/Partial/No
P4	Is the type of study clearly identified and justified?	Yes/Partial/No
P5	Does the study establish a well-defined purpose regarding the integration of mathematical competencies and critical thinking in secondary education?	Yes/Partial/No
P6	Are standards or reference frameworks on the teaching of mathematical competencies and the development of critical thinking in secondary education identified?	Yes/Partial/No
P7	Does the study refer to pedagogical models, teaching approaches, or theories that support the integration of mathematical competencies and critical thinking?	Yes/Partial/No
P8	Are the key aspects to be considered in the teaching of mathematical competencies to strengthen critical thinking in secondary students established?	Yes/Partial/No
P9	Are data presented on the evaluation of strategies or teaching approaches used to develop mathematical competencies and critical thinking in secondary education?	Yes/Partial/No
P10	Are the research questions formulated in the study aimed at providing clear answers and solutions to the issue of integrating mathematical competencies and critical thinking in secondary education?	Yes/Partial/No

A minimum threshold of ≥7 out of 10 points was established to ensure acceptable methodological quality, while avoiding overly restrictive exclusion. While this cut-off is not universal, it has been used in previous educational reviews. Future studies are encouraged to complement this approach with sensitivity analyses and more graduated scoring systems to better capture methodological nuances. Table 2 presents the applied questions, the evaluation criteria, and the scoring scale used for selection: “yes” = 1 point, “partially” = 0.5 points, and “no” = 0 points.

To ensure methodological rigor while respecting the epistemological and disciplinary characteristics of educational research, the quality appraisal was guided by the AMSTAR 2 checklist ( Shea et al., 2017). Although this instrument was originally developed for systematic reviews of randomized and non-randomized health interventions, it was employed here as a methodological framework to ensure coherence and traceability in the evaluation process. A final quantitative rating was not produced; as certain indicators are not directly applicable to the educational field. The full document containing the selected AMSTAR 2 items is available in open access on Figshare ( https://doi.org/10.6084/m9.figshare.30490835).

Table 3 summarizes the key characteristics of the 24 studies included in this systematic review. It presents information on the authors and publication year, article title, methodological approach, population/sample/studies analyzed, country of origin, and the quality score assigned according to the adapted evaluation matrix.

**Table 3.** Articles included in the review.

Authors (Year)	Title	Type of methodology/Study design	Population/ Sample/Studies analyzed	Country	Score
Chacón-Cueva et al. (2023)	Aprendizaje basado en problemas para desarrollar el pensamiento crítico en estudiantes de secundaria – 2023	Quantitative, quasi-experimental design.	92 students (50 in the experimental group and 42 in the control group)	Peru	7
Sutama et al. (2022)	Collaborative mathematics learning management: Critical thinking skills in problem solving	Qualitative, descriptive ethnographic study.	34 participants (1 school principal, 3 mathematics teachers, and 30 students)	Indonesia	10
Llerena-Aguilar et al. (2023)	Metodologías innovadoras basadas en el aprendizaje basado en retos y problemas: una mirada a la mejora de la competencia lógico matemática	Applied research, mixed-methods approach (quantitative-qualitative).	200 students	Ecuador	9
Pinos-Vargas et al. (2024)	El Impacto del Aprendizaje Basado en Problemas (ABP) en el Desarrollo del Pensamiento Matemático Crítico en Estudiantes de Educación Básica	Theoretical–analytical review with constructivist perspective.	44 documents analyzed	Ecuador	8.5
Jiménez-Cortes & Vesga-Bravo (2022)	Fortalecimiento del pensamiento crítico en el aula de matemáticas: una experiencia en pandemia	Qualitative, participatory action research.	18 students	Colombia	10
Alvis-Puentes et al. (2019)	Los ambientes de aprendizaje reales como estrategia pedagógica para el desarrollo de competencias matemáticas en estudiantes de básica secundaria	Qualitative, interpretive approach (critical mathematics education).	Secondary school students (grade 9). The number of students is not specified.	Colombia	10
Castro-Valle et al. (2023)	Estrategia aprendizaje basado en proyectos para desarrollar el pensamiento crítico en estudiantes de secundaria	Quantitative, quasi-experimental design.	60 students	Peru	7.5
Hilario (2021)	Aprendizaje basado en proyectos mediados por Tic para desarrollar competencias matemáticas en estudiantes de secundaria	Quantitative, quasi-experimental design.	57 students (30 experimental group, 27 control)	Peru	10
Wahono et al. (2020)	Evidence of STEM enactment effectiveness in Asian student learning outcomes	Systematic review and meta-analysis.	54 studies selected from an initial sample of 4768 articles, after quality and relevance screening	Taiwan	7
Viro & Joutsenlahti (2020)	Learning mathematics by project work in secondary school	Quantitative, multiple-case quasi-experimental study.	117 students (59 in coordinate project, 58 in statistics project)	Finland	10
Val Fernández (2023)	Matemáticas con Fermat. Propuesta práctica para potenciar la toma de decisiones en las aulas de secundaria	Theoretical–practical proposal for educational innovation.	Secondary school students; the number of participants is not specified because it was not empirically applied.	Spain	8
Moreira et al. (2024)	Gamification for learning mathematics in secondary school: Most effective gamified strategies to motivate students and improve their performance in mathematics	Theoretical review of gamification in mathematics.	15 studies selected from an initial sample of 150 articles, after quality and relevance screening	Spain	7
Urdanivia et al. (2023)	Science and inquiry-based teaching and learning: a systematic review	Systematic review.	51 studies selected from an initial sample of 700 articles, after quality and relevance screening	Peru	7
Farfán-Pimentel et al. (2022)	Quizizz en el desarrollo de competencias matemáticas en estudiantes de secundaria: Una revisión teórica	Theoretical review.	168 studies analyzed	Peru	7
Curto et al. (2019)	Student Assessment of the Use of Kahoot in the Learning Process of Science and Mathematics	Quantitative, descriptive quasi-experimental design.	35 students	Spain	7
Del Cerro & Morales (2021)	Application in Augmented Reality for Learning Mathematical Functions: A Study for the Development of Spatial Intelligence in Secondary Education Students	Mixed-methods, quasi-experimental design.	48 students	Spain	9
Alegre et al. (2019)	Peer tutoring and mathematics in secondary education: literature review, effect sizes, moderators, and implications for practice	Systematic review and meta-analysis.	42 studies selected from an initial sample of 143 articles, after quality and relevance screening	Spain	7,5
Hillmayr et al. (2020)	The potential of digital tools to enhance mathematics and science learning in secondary schools: A context-specific meta-analysis	Meta-analysis of empirical studies (2000–2019).	16 studies selected from an initial sample of 6572 articles, after quality and relevance screening	Germany	8
McLure et al. (2022)	What do integrated STEM projects look like in middle school and high school classrooms? A systematic literature review of empirical studies of iSTEM projects	Systematic review of empirical studies on integrated STEM projects.	35 studies selected from an initial sample of 221 articles, after quality and relevance screening	Australia	7
Saldarriaga-Cantos et al. (2024)	Desarrollo del pensamiento crítico en la ejecución de proyectos interdisciplinares basados en tecnologías de la información y comunicación	Mixed, descriptive-correlational, inductive-deductive design.	175 participants (165 students, 7 teachers, 2 principals, and 1 guidance counselor)	Ecuador	10
Cruz et al. (2022)	Project-Based Learning Methodology as a Promoter of Learning Math Concepts: A Scoping Review	Scoping review.	17 empirical studies	Portugal	7,5
Mayorga-Aguirre et al. (2024)	Educación STEM: Fomentando el Pensamiento Crítico y la Innovación en las Aulas	Mixed-methods approach (quantitative-qualitative).	60 participants (40 students and 20 teachers)	Ecuador	10
Maskur et al. (2022)	La comparación del enfoque STEM y el modelo de aprendizaje SSCS para la escuela secundaria basado en el plan de estudios K-13: el impacto en la capacidad de pensamiento creativo y crítico	Quantitative, quasi-experimental design.	125 secondary students (65 in Group I, 60 in Group II)	Indonesia	9
Ordóñez-Barberán & Sánchez-Godoy (2024)	Estrategias metacognitivas para la enseñanza de las matemáticas en educación secundaria	Qualitative, descriptive design.	45 documents analyzed	Ecuador	10

This synthesis supports the thematic categorization presented in the Results section and contextualizes the scope and relevance of each analyzed study.

## 3. Results

As detailed in Table 3 ( Section 2, Methods), the 24 studies included in this systematic review exhibit significant diversity in their methodological designs, geographic contexts, and target populations. This heterogeneity provides the empirical foundation for the thematic analysis presented below, which is structured around the three research questions (RQ1, RQ2, and RQ3).

To enhance readability and ensure consistency in the interpretation of the results, all percentages reported in this section were rounded to the nearest whole number, following the guidelines of the Publication Manual of the American Psychological Association (7th ed.; American Psychological Association, 2020, §6.36), which recommends expressing percentage values with a level of precision appropriate to the size and nature of the dataset.

Interest in integrating mathematical competencies and critical thinking in secondary education has shown steady growth between 2019 and 2025. Over the past three years (2022–2024), scientific output increased by 67%, with 2023 emerging as the most prolific year, accounting for six studies, 25% of the total. These data reflect a sustained upward trend in the field, responding to the educational imperative of fostering higher-order thinking skills in diverse school contexts.

The peak in publications observed in 2023 can be attributed to the maturation of post-pandemic educational innovation projects, increased institutional funding for digital transformation, and heightened policy emphasis on transversal competencies. This pattern aligns with global reports indicating a surge in research on critical thinking and competency-based education following the pandemic ( UNESCO, 2022; OECD, 2023).

In terms of geographic distribution, 50% of the studies were conducted in Latin America, particularly in Ecuador and Peru (five studies each) and Colombia (two studies). Europe accounts for 29% of the total, with research conducted in Spain (four studies), Portugal (one), Germany (one), and Finland (one). Asia contributes 13%, represented by two studies from Indonesia and one from Taiwan, while Oceania is represented by a single study (4%) conducted in Australia. The absence of studies from Africa and North America highlights a limitation in the global generalizability of the findings and reveals a geographic gap that future research could address.

From a methodological perspective (see Table 3), the analyzed studies display a wide range of approaches. Quantitative designs predominate, comprising nine studies (38%), mostly quasi-experimental or descriptive in nature, aimed at evaluating the effects of active learning methodologies or digital tools on mathematics learning ( Chacón-Cueva et al., 2023; Castro-Valle et al., 2023; Hilario, 2021; Curto et al., 2019; Del Cerro & Morales, 2021; Maskur et al., 2022; Saldarriaga-Cantos et al., 2024; Viro & Joutsenlahti, 2020; Hillmayr et al., 2020).

Three studies (13%) applied a mixed-methods approach, combining quantitative and qualitative analyses to gain a comprehensive understanding of the pedagogical impact of the implemented strategies ( Del Cerro & Morales, 2021; Mayorga-Aguirre et al., 2024; Saldarriaga-Cantos et al., 2024).

Four studies (17%) employed qualitative methodologies, ethnographic, field-based, or participatory action research, focused on classroom experiences, teachers’ perspectives, and collaborative learning processes ( Sutama et al., 2022; Jiménez-Cortés & Vesga-Bravo, 2022; Alvis-Puentes et al., 2019; Ordóñez-Barberán & Sánchez-Godoy, 2024).

Finally, eight studies (33%) correspond to systematic, theoretical, or exploratory reviews, including meta-analyses, scoping reviews, and documentary analyses that provide a broad overview of the state of the art and conceptual trends in the integration of mathematical competencies and critical thinking ( Pinos-Vargas et al., 2024; Farfán-Pimentel et al., 2022; Moreira et al., 2024; Urdanivia et al., 2023; Alegre et al., 2019; McLure et al., 2022; Wahono et al., 2020; Val Fernández, 2023).

The most relevant results of this study are presented below, based on each of the research questions posed (RQ1, RQ2, and RQ3).

### 3.1 RQ1. How are mathematical competencies and critical thinking integrated into secondary education?

The analysis of the 24 studies examined reveals that 87% (21 out of 24) explicitly integrate both dimensions, mathematical competence and critical thinking, through active, student-centered methodologies, including Problem-Based Learning (PBL), Project-Based Learning (PjBL), STEM/STEAM education, the use of educational technologies and gamification, as well as metacognitive strategies and peer tutoring.

Thirteen percent correspond (3 studies) correspond to theoretical or systematic reviews ( Pinos-Vargas et al., 2024; Moreira et al., 2024; Val Fernández, 2023), which address the convergence between both constructs from pedagogical and epistemological perspectives, providing solid conceptual frameworks but lacking direct empirical application.

Overall, these results indicate that the integration between critical thinking and mathematical competence relies mainly on active and experimental approaches aimed at developing logical reasoning, creativity, and metacognitive reflection.

Problem-Based Learning (PBL) was documented in six studies (25%) ( Chacón-Cueva et al., 2023; Sutama et al., 2022; Llerena-Aguilar et al., 2023; Jiménez-Cortes & Vesga-Bravo, 2022; Alvis-Puentes et al., 2019; Castro-Valle et al., 2023), where real-world problem solving was used to foster logical reasoning, argumentation, and metacognitive awareness. The designs were mainly quasi-experimental and participatory, with samples ranging from 60 to 200 secondary school students in Peru, Ecuador, Colombia, and Indonesia.

Project-Based Learning (PjBL) was reported in five studies (21%) ( Hilario, 2021; Viro & Joutsenlahti, 2020; Val Fernández, 2023; Cruz et al., 2022; Castro-Valle et al., 2023), focusing on interdisciplinary integration and cognitive transfer. These studies employed long-term collaborative tasks and performance rubrics designed to assess students’ ability to argue, plan, and apply mathematical concepts to contextualized situations.

Technological integration emerged as another relevant approach in five studies (21%) ( Curto et al., 2019; Del Cerro & Morales, 2021; Farfán-Pimentel et al., 2022; Hillmayr et al., 2020; Saldarriaga-Cantos et al., 2024), which implemented tools such as Kahoot, Quizizz, and Augmented Reality. These resources were used to strengthen active participation, analytical thinking, and critical performance assessment, demonstrating the mediating role of technology in reflective mathematics learning.

STEM/STEAM education was identified in four studies (17%) ( Mayorga-Aguirre et al., 2024; Maskur et al., 2022; McLure et al., 2022; Wahono et al., 2020), where the integration of science, technology, engineering, and mathematics was proposed as a strategy to foster computational thinking, creativity, and logical reasoning. These studies, using quantitative, mixed, and meta-analytical approaches, converge in highlighting the potential of the STEM framework to bridge critical thinking with complex problem-solving.

Other complementary strategies include gamification ( Moreira et al., 2024; Urdanivia et al., 2023), cooperative learning ( Alegre et al., 2019), and metacognitive strategies ( Ordóñez-Barberán & Sánchez-Godoy, 2024), each reported in one study (4%). These approaches emphasize cognitive self-regulation, peer tutoring, and metacognitive reflection as essential components in developing critical mathematical thinking.

As summarized in Table 4, the methodologies identified, although diverse in implementation, share a common goal: to train students capable of analyzing, reasoning, and transferring mathematical knowledge to complex, contextualized situations. However, there remains a lack of validated psychometric and evaluative models that would allow for standardized measurement of their impact in educational contexts, representing a relevant direction for future empirical research.

**Table 4.** Methodological strategies used in the analyzed studies.

Strategy/Method	Studies	Number of publications	Percentage
Problem-Based Learning (PBL)	Chacón-Cueva et al. (2023); Sutama et al. (2022); Llerena et al. (2023); Pinos et al. (2024); Jiménez-Cortes & Vesga-Bravo (2022); Alvis-Puentes et al. (2019)	6	25%
Project-Based Learning (PjBL)	Castro-Valle et al. (2023); Hilario (2021); Viro & Joutsenlahti (2020); Val-Fernández (2023); Cruz et al. (2022)	5	21%
Use of Educational Technologies (Kahoot, Quizizz, AR)	Farfán-Pimentel et al. (2022); Curto et al. (2019); del Cerro & Morales (2021); Hillmayr et al. (2020); Saldarriaga et al. (2024)	5	21%
STEM Education	Mayorga-Aguirre et al. (2024); Maskur et al. (2022); McLure et al. (2022); Wahono et al. (2020)	4	17%
Gamification	Moreira et al. (2024); Urdanivia et al. (2023)	2	8%
Cooperative Learning	Alegre et al. (2019)	1	4%
Metacognitive Strategies	Ordóñez-Barberán & Sánchez-Godoy (2024)	1	4%

### 3.2 RQ2. What is the impact of integrating mathematical competencies and critical thinking on academic performance and the development of cognitive skills in secondary education students?

The analysis of 22 empirical studies shows that the integration of mathematical competence and critical thinking consistently produces positive effects on both academic performance and the development of higher-order cognitive skills among secondary-school students. The most frequent improvements were observed in conceptual understanding, logical reasoning, creativity, self-regulation, and problem-solving. However, the magnitude of the effects varied according to methodological design, implementation context, and intervention length.

An average medium effect size (Cohen’s d ≈ 0.55) was reported in approximately 86% of the studies, although several lacked complete data on sample size, statistical tests, or intervention duration, which introduces a potential publication bias. Two non-empirical studies ( Pinos-Vargas et al., 2024; Val Fernández, 2023) provided theoretical and conceptual frameworks, highlighting the need for validated psychometric models and longitudinal designs to measure the relationship between critical thinking and mathematical achievement more rigorously.

• **Impact on academic performance**

Five studies on Problem-Based Learning (PBL) ( Chacón-Cueva et al., 2023; Sutama et al., 2022; Llerena-Aguilar et al., 2023; Jiménez-Cortes & Vesga-Bravo, 2022; Alvis-Puentes et al., 2019) reported average grade increases of 10–15%, with moderate effects (d ≈ 0.5–0.6) on logical reasoning and metacognitive reflection. Most interventions, lasting between four and twelve weeks with samples of 60–200 students, used digital simulation tools, online questionnaires, and virtual learning environments to foster autonomous and collaborative problem-solving in mathematics.

Project-Based Learning (PjBL), analyzed in four studies ( Hilario, 2021; Castro-Valle et al., 2023; Viro & Joutsenlahti, 2020; Cruz et al., 2022), generated 10–18% increases in academic performance (p < .05), enhancing planning, mathematical communication, and teamwork. These projects integrated digital tools such as Google Classroom, Wordwall, and Educaplay to manage virtual classrooms, present final outputs, and enable formative self-assessment.

Within the STEM/STEAM framework ( Mayorga-Aguirre et al., 2024; Maskur et al., 2022; McLure et al., 2022; Wahono et al., 2020), moderate-to-high effect sizes (d ≈ 0.6–0.8) were associated with the use of virtual labs, 3D modeling software, and coding platforms to address interdisciplinary problems. These approaches fostered creativity, logical reasoning, and evidence-based decision-making through six-to-ten-week interventions supported by pre-post statistical analyses (e.g., ANOVA).

• **Impact on cognitive development**

Seven studies integrating digital technologies and gamification ( Curto et al., 2019; Del Cerro & Morales, 2021; Farfán-Pimentel et al., 2022; Hillmayr et al., 2020; Saldarriaga-Cantos et al., 2024; Urdanivia et al., 2023; Moreira et al., 2024) reported gains in motivation, autonomy, and critical-thinking skills (d ≈ 0.4–0.6). Gamified environments such as Quizizz, Kahoot, and augmented-reality applications provided immediate feedback, formative assessment, and playful learning experiences that improved self-efficacy and conceptual understanding.

Collaborative and metacognitive approaches ( Alegre et al., 2019; Ordóñez-Barberán & Sánchez-Godoy, 2024) revealed progress in self-regulation, communication, and reflective reasoning, though without statistical quantification of the effect size.

Overall, these findings demonstrate a direct relationship between the pedagogical use of interactive technologies and the development of critical thinking within mathematical contexts. Table 5 summarizes the empirical evidence, specifying the methodological approaches, technologies used, and the reported effects on academic performance and cognitive development.

**Table 5.** Empirical evidence on the impact of integrating mathematical competence and critical thinking (RQ2).

Study	Main findings and cognitive impact	Effect size
Chacón-Cueva et al. (2023)	PBL reduced low achievement from 62% to 8%; improved logical reasoning and metacognition using online problem simulations.	d ≈ 0.5 – 0.6
Sutama et al. (2022)	PBL enhanced analytical thinking and autonomy through contextual mathematical problems in small-group settings.	d ≈ 0.5
Llerena-Aguilar et al. (2023)	PBL improved logical-mathematical competence; reported very high effect (Cohen’s d = 5.15, likely overestimated).	—
Jiménez-Cortes & Vesga-Bravo (2022)	PBL strengthened argumentation and reasoning using real-context problems with collaborative reflection.	d ≈ 0.6
Alvis-Puentes et al. (2019)	PBL improved critical argumentation and ethical decision-making in math education.	d ≈ 0.5
Hilario (2021)	PjBL + digital tools increased math test scores by ~18%; improved planning and communication.	p < .05
Castro-Valle et al. (2023)	PjBL raised grades by ~10%; 92% of students reached medium cognitive levels in argumentation.	—
Viro & Joutsenlahti (2020)	Interdisciplinary PjBL improved modeling and reflective reasoning with STEM integration.	d ≈ 0.6
Cruz et al. (2022)	PjBL fostered teamwork, creativity, and applied reasoning in contextualized problems.	—
Mayorga-Aguirre et al. (2024)	STEM boosted creativity (+20% in grades vs 10% control); 75% reported improved problem-solving.	d ≈ 0.7
Maskur et al. (2022)	STEM increased computational thinking and logic through coding-based activities.	d ≈ 0.6
McLure et al. (2022)	Meta-review: theoretical–practical gap persists; calls for stronger empirical validation.	—
Wahono et al. (2020)	STEM enhanced reasoning and collaboration in engineering-math integration.	d ≈ 0.8
Curto et al. (2019)	Kahoot/Quizizz improved motivation and attention; students scored +12% on average.	d ≈ 0.5
Del Cerro & Morales (2021)	Augmented Reality improved spatial reasoning and conceptual understanding in geometry.	d ≈ 0.4
Farfán-Pimentel et al. (2022)	Gamified mobile apps enhanced autonomy and self-evaluation in algebra learning.	d ≈ 0.6
Hillmayr et al. (2020)	Meta-analysis on EdTech: moderate positive effects on math achievement.	d ≈ 0.5
Saldarriaga-Cantos et al. (2024)	Digital learning objects improved creativity and analytical reasoning.	d ≈ 0.6
Urdanivia et al. (2023)	Gamification strengthened conceptual understanding and evidence-based reasoning.	d ≈ 0.5
Moreira et al. (2024)	Mixed outcomes: higher motivation, uneven academic gains; emphasizes need for robust design.	—
Alegre et al. (2019)	Cooperative learning enhanced socio-emotional and logical-mathematical skills.	—
Ordóñez-Barberán & Sánchez-Godoy (2024)	Metacognitive strategies improved reflection and self-regulation through peer tutoring.	—

**Note.** Effect sizes were interpreted following Cohen (2013): small ≈ 0.2, medium ≈ 0.5, large ≥ 0.8. Percentages were rounded to the nearest whole number ( American Psychological Association, 2020, § 6.44). The symbol “—” indicates that the study does not report quantitative data on the effect size (e.g., *p*-values, *t*, ANOVA, or Cohen’s *d*), although it provides qualitative or descriptive evidence of impact.

### 3.3 RQ3. What are the main barriers and challenges faced in integrating critical thinking into mathematics teaching at the secondary level?

The implementation of critical thinking in mathematics teaching in secondary education faces various structural and pedagogical barriers that hinder its effective integration and, consequently, the holistic development of students. Based on the systematic literature review, five main challenges were identified that obstruct its application in the classroom:

• **Limited teacher preparation and resistance to change.** One of the most frequent issues identified in the reviewed studies is the lack of teacher training in active methodologies and their resistance to implementing them. Although approaches such as Problem-Based Learning (PBL) and Project-Based Learning (PjBL) have proven effective in developing critical thinking ( Sutama et al., 2022; McLure et al., 2022), their application in educational practice remains limited. Factors such as insufficient pedagogical training, reluctance to innovate, and challenges in curricular planning contribute to the persistence of traditional models focused on unidirectional content transmission. This didactic rigidity restricts the development of complex cognitive skills such as analysis, argumentation, and critical evaluation ( Castro-Valle et al., 2023; Chacón-Cueva et al., 2023; Alegre et al., 2019; Cruz et al., 2022).
• **Limited student research culture.** Another identified challenge is students’ limited readiness and ability to engage in research processes. Some studies note that students often engage in mechanical reading and lack deep comprehension skills, negatively affecting their ability to interpret information, formulate hypotheses, analyze data, and coherently communicate findings ( Chacón-Cueva et al., 2023; Saldarriaga-Cantos et al., 2024). This weakness directly impacts the possibility of applying well-founded critical thinking ( Castro-Valle et al., 2023).
• **Rigid curriculum and low contextualization.** Several studies agree that current curricula tend to be rigid and standardized, with limited openness to interdisciplinary approaches that link mathematics with other fields of knowledge or real-world situations ( Llerena et al., 2023). This curricular disconnection reduces students’ ability to transfer and apply mathematical knowledge to authentic and meaningful contexts, thus limiting the development of critical skills necessary to tackle complex problems ( Viro & Joutsenlahti, 2020; Alvis-Puentes et al., 2019).
• **Deficiencies in formative assessment and feedback.** The lack of effective continuous assessment mechanisms and timely feedback constitutes an additional barrier. Studies such as Val-Fernández (2023) highlight that the absence of contextualized evaluative criteria and personalized feedback prevents students from recognizing their mistakes and progress, thereby hindering their cognitive development. Furthermore, other works indicate that assessments focused solely on quantitative results, combined with limited classroom time, encourage rote repetition and reduce opportunities for reflective and autonomous learning ( Viro & Joutsenlahti, 2020; McLure et al., 2022).
• **Lack of contextualization in mathematics learning.** Finally, several authors emphasize that the disconnect between mathematical content and real-life contexts contributes to student demotivation, hindering their engagement in cognitively demanding activities ( Moreira et al., 2024). This lack of practical contextualization reinforces the perception of mathematics as abstract and disconnected from everyday reality, which inhibits the activation of critical thinking, an ability that requires the application of knowledge in authentic, challenging, and meaningful situations.

## 4. Discussion

This systematic review critically examined the integration of mathematical competence and critical thinking in secondary education, highlighting both the impact of pedagogical strategies and the contextual barriers affecting their implementation. The findings confirm that this integration is currently in a stage of consolidation, characterized by an increasing use of active methodologies mediated by technology. However, beyond validating the effectiveness of approaches such as Problem-Based Learning (PBL), Project-Based Learning (PjBL), STEM/STEAM, or gamification, this discussion seeks to understand why these strategies work, under what conditions they generate impact, and what their structural and contextual limitations are.

From a theoretical perspective, the findings reveal three common pedagogical mechanisms that explain the effectiveness of these strategies: the contextualization of knowledge through real problem-solving, technological and symbolic mediation, and guided and reflective collaboration. These principles, derived from constructivism ( Piaget, 1972), critical pedagogy ( Freire, 1970), and sociocultural learning theory ( Vygotsky, 1978), conceive learning as an active, situated, and transformative process. In line with this, studies such as those by Chacón-Cueva et al. (2023), Hilario (2021), and Sutama et al. (2022) demonstrate that PBL and PjBL foster logical reasoning, argumentation, and metacognition when implemented in flexible environments with reflective guidance. Comparable findings were reported by Soia et al. (2024), who showed that project-based learning fosters both critical thinking and teamwork skills among future mathematics and computer-science teachers, supporting the collaborative and reflective principles identified in this review. Similarly, Li and Oon (2024) and Khalil et al. (2023) show that critical thinking emerges when learning encourages dialogue, autonomy, and the co-construction of knowledge.

Some studies, however, warn of significant limitations. Trisnani et al. (2024) and Moreira et al. (2024) report that in contexts characterized by rigid curricula or limited teacher training, PBL implementation can devolve into a sequence of activities lacking reflective depth. This finding aligns with Le (2024), who notes that active methodologies may fail if not supported by coherent institutional structures. Furthermore, the lack of explicit theoretical alignment between empirical designs and epistemological frameworks remains a cross-cutting weakness. This disconnect, also identified by Sánchez-García and Reyes-de-Cózar (2025) and Van der Zanden et al. (2020), limits the replicability and transferability of findings and underscores the need for more robust methodological models that integrate instructional design, psychometric evaluation, and longitudinal analysis.

The STEM/STEAM approach has emerged as an integrative strategy that combines logical reasoning, creativity, and problem-solving in interdisciplinary contexts ( Mayorga-Aguirre et al., 2024; Maskur et al., 2022; Pratiwi et al., 2025). Recent quasi-experimental evidence also shows that STEM-based learning produces significantly higher gains in mathematical critical thinking compared to conventional instruction, reinforcing its value as an integrative pedagogical model ( Pratiwi et al., 2025). This result is consistent with the findings of Margot and Kettler (2019), who argue that STEM facilitates the transfer of mathematical knowledge to real-world contexts. Brandsæter and Berge (2025) further extend this perspective by demonstrating that programming-based activities effectively strengthen mathematical competence through computational reasoning, highlighting the potential of digital environments to promote mathematical fluency and problem-solving. However, the review reveals significant contrasts across regions and institutional levels. While Yohannes et al. (2024) and Er (2024) report sustained improvements in cognitive transfer and motivation, Suherman et al. (2022) and Kalinin and Toropova (2024) warn that gender gaps, limited curricular interdisciplinarity, and unequal teacher preparation condition the outcomes. These differences confirm that the effectiveness of STEM is context-dependent: its benefits do not derive solely from the pedagogical model itself but also from the institutional conditions that support it, as observed by Hillmayr et al. (2020) and Duma et al. (2024).

Findings related to technological mediation represent another central axis in the integration of competencies. Digital tools such as Kahoot, Quizizz, GeoGebra, and augmented reality function as symbolic mediators that promote immediate feedback, active participation, and self-regulated learning ( Curto et al., 2019; Del Cerro & Morales, 2021). Nevertheless, several studies caution that the instrumental use of these technologies does not necessarily guarantee deep learning. Nurdin et al. (2023) found that PBL environments supported by Google Sites significantly enhanced students’ mathematical critical thinking, confirming that digital scaffolding amplifies the reflective and metacognitive components of active learning. Nashrullah et al. (2023) and Saldarriaga-Cantos et al. (2024) similarly noted that teachers’ digital competence and structured pedagogical design are essential to transforming technology from a motivational tool into a medium for higher-order reasoning. This divergence aligns with McLure et al. (2022), who recommend a reflective and situated use of technological tools that integrates authentic assessment and collaborative learning criteria.

The reviewed studies identify a recurring set of structural barriers: rigid curricula, insufficient teacher training, weak student research culture, and the absence of instruments for assessing higher-order skills ( Castro-Valle et al., 2023; Cruz et al., 2022; Kintoko et al., 2024). These limitations do not operate independently but interact, creating a “funnel effect” ( Nicholus et al., 2023) in which methodological innovations are diluted by inflexible institutional structures or memorization-based assessments. Accordingly, PBL and PjBL tend to fail in environments with rigid timetables and limited curricular autonomy, while the lack of authentic assessment obscures progress in critical and logical reasoning.

Another critical finding is the prevalence of publication bias: most of the reviewed studies report positive effects, while mixed or null results are rare ( Moreira et al., 2024; Le, 2024). This pattern, also highlighted by Hillmayr et al. (2020) and McLure et al. (2022), underscores the need to promote more transparent research practices, including sensitivity analyses, open data, and cross-context replication. Encouraging longitudinal and multicultural studies would help prevent the overestimation of effectiveness and yield a more nuanced understanding of the mediating factors that influence success.

Based on the evidence analyzed, three key practical implications can be identified. The first concerns strengthening initial and continuing teacher training focused on critical reflection, formative assessment, and the pedagogical use of interactive technologies ( Hillmayr et al., 2020; Duma et al., 2024). The second highlights the need to design flexible, interdisciplinary curricula that integrate mathematics with other domains to foster cognitive transfer, creativity, methodological experimentation, and authentic assessment ( Er, 2024). The third relates to implementing institutional policies for educational innovation that ensure technological infrastructure, pedagogical support, and context-sensitive evaluation systems to apply these strategies equitably and sustainably ( Peralta et al., 2025; Melo-López et al., 2025; Jaramillo-Mediavilla et al., 2024). These recommendations, consistent with the proposals of United Nations Educational, Scientific and Cultural Organization ( UNESCO, 2023) and the Organisation for Economic Co-operation and Development ( OECD, 2022), aim to transcend the academic sphere and inform public policy and teacher development initiatives.

Finally, it is important to acknowledge the limitations of this review. The geographic concentration of studies in Latin America and Southern Europe introduces a contextual bias that restricts the generalizability of the findings. Further research is needed in underrepresented regions, such as Africa, Central Asia, and the Middle East, to compare outcomes and advance toward a global understanding of the phenomenon. Future studies should prioritize intercultural and collaborative approaches supported by validated psychometric models to strengthen comparability and sustainability.

Overall, this review provides a critical synthesis that integrates pedagogical, technological, and structural evidence concerning critical mathematical education, contributing to the construction of an educational action framework grounded in recent empirical evidence. Its impact will depend on the ability of educational systems to generate structural, policy, and training conditions that ensure its sustainable and equitable implementation.

## 5. Conclusions

This systematic review demonstrates that the integration of mathematical competencies and critical thinking in secondary education is still in a stage of conceptual consolidation rather than full pedagogical maturity. The analysis of 24 studies (2019–2025) reveals that while active methodologies, such as PBL, PjBL, STEM/STEAM, and gamification, consistently produce positive cognitive and motivational outcomes, their success depends strongly on contextual variables such as institutional support, teacher expertise, and curriculum flexibility.

The main contribution of this review lies not in reaffirming the effectiveness of these strategies, but in identifying the underlying mechanisms and systemic conditions that enable their impact: authentic problem-solving and contextualization of learning; guided collaboration that cultivates metacognitive reflection; and technological mediation that promotes autonomy and formative assessment. These principles form the basis of an emerging integrative pedagogical framework linking critical reasoning, creativity, and digital fluency in mathematics education.

At the same time, this synthesis exposes persistent structural inequities: most successful interventions occur in well-resourced schools with high teacher autonomy. Thus, replicability in less favorable contexts remains uncertain. For policymakers and practitioners, this review highlights the need to move beyond isolated innovations and toward systemic reform, strengthening teacher professional learning communities, investing in digital infrastructure, and embedding authentic assessment of reasoning and argumentation in curricula.

Ultimately, the integration of mathematical and critical thinking competencies should be viewed not as a set of discrete strategies, but as a transformative capacity of educational systems to generate equitable and evidence-informed environments where students learn to think mathematically and critically about real-world challenges.

### 5.1 Limitations

This review faces several methodological and contextual limitations that affect the interpretation and generalization of its findings.

Scope and search bias. The review included publications indexed in Scopus, Web of Science, SciELO, and Dialnet, omitting databases such as ERIC, ProQuest, and Google Scholar. This restricted scope may have excluded relevant studies, particularly those published in educational or regional journals not indexed in these repositories. Moreover, limiting the search to English and Spanish introduced a language bias that likely underrepresents research from Asia and Africa.

Publication and selection bias. Only peer-reviewed journal articles were considered, excluding gray literature, dissertations, and conference papers. This decision may have contributed to a positive publication bias, as studies reporting null or negative results are less likely to be published. Although the screening process ensured methodological rigor (κ = 0.83), the absence of gray literature may overestimate the effectiveness of the analyzed interventions.

Heterogeneity and contextual imbalance. The included studies display wide variation in design, theoretical grounding, and operational definitions of mathematical competence and critical thinking. This heterogeneity precludes meta-analytic synthesis and limits cross-study comparability. Furthermore, geographical concentration in Latin America and Southern Europe reduces global representativeness. The underrepresentation of African, Middle Eastern, and South Asian contexts constrains external validity and the ability to identify cultural moderators.

Temporal and evidential limitations. Given that most studies were published between 2020 and 2024, the field remains emergent. The small sample (n = 24) and limited number of longitudinal or randomized studies suggest that conclusions about causality remain preliminary. Future replications and standardized designs are required to strengthen reliability.

Despite these limitations, this review successfully synthesizes the available empirical evidence in a rigorous and systematic manner, providing a clear and updated view of the current state of knowledge in this field and guiding future lines of research.

### 5.2 Recommendations for future research

Future investigations should transcend descriptive approaches and focus on analytical, comparative, and longitudinal designs that allow causal inference about the relationship between critical thinking and mathematical competencies. Several priorities emerge from this review:

Digital and AI-based learning innovations. The promising role of interactive technologies (Kahoot, GeoGebra, AR) calls for deeper evaluation of AI-assisted learning environments and adaptive feedback systems that scaffold metacognitive reflection in mathematics.

Equity and inclusion. Empirical studies should examine how gender, socioeconomic status, disability, and linguistic diversity mediate the relationship between pedagogy and critical thinking outcomes. Comparative research across educational systems can reveal which interventions promote equitable access to critical mathematical literacy.

Teacher professional learning and policy alignment. Rigorous experimental and design-based studies are needed to evaluate models such as lesson study, professional learning communities, and reflective coaching, linking teacher development with measurable student outcomes.

Assessment innovation. The field would benefit from validated instruments for assessing critical thinking within mathematical contexts, including authentic performance tasks, scenario-based reasoning, and computational thinking rubrics.

Global and intercultural expansion. Future research must include underrepresented regions, Africa, South Asia, and the Middle East, to address geographical bias and build a more globally representative evidence base. Cross-cultural meta-analyses could elucidate how sociocultural variables influence the effectiveness of active methodologies.

These future research directions aim to contribute to improving the quality of education, preparing students to face the challenges of the 21st century in a reflective, creative, and ethical manner.

## Data Availability

Figshare: PRISMA-based systematic literature review (2019–2025) on mathematical competencies and critical thinking in secondary education. https://doi.org/10.6084/m9.figshare.c.7946345.v3 ( Alvarez et al., 2025a). The project contains the following underlying data:
•
SRL Mathematical skills and critical thinking.xlsx – This file compiles all the data used and analyzed in each phase of the systematic review conducted in accordance with PRISMA 2020. It includes the records identified in the searches, the removal of duplicates, the articles selected during the screening process, the studies evaluated in the eligibility stage, and those ultimately included in the analysis. In addition, it contains the individual item-level responses, total scores, and the analysis of each research question (RQ) related to mathematical skills and critical thinking. • Flow diagram SRL mathematical skills and critical thinking.docx – Contains the PRISMA 2020 flow diagram detailing the study selection process, including the records identified, excluded, assessed for eligibility, and ultimately included in the systematic review. •

PRISMA_2020_checklist.pdf – Contains the PRISMA 2020 checklist documenting compliance with all required reporting items for the systematic review. Each underlying dataset is available at the following Figshare records:
• Figshare: SRL Mathematical skills and critical thinking. https://doi.org/10.6084/m9.figshare.29635733.v2 ( Alvarez et al., 2025b). • Figshare: Flow diagram SRL mathematical skills and critical thinking. https://doi.org/10.6084/m9.figshare.29825183.v1 ( Alvarez et al., 2025c). • Figshare: PRISMA_2020_checklist. https://doi.org/10.6084/m9.figshare.29825231.v1 ( Alvarez et al., 2025d). Data are available under the terms of the Creative Commons Attribution 4.0 International license (CC-BY 4.0).
