# Supplementary material for: Mathematical Competencies and Critical Thinking in Secondary Education: A PRISMA-Based Systematic Review (2019–2025)
Source: F1000Res. 2026 Jan 21;14:1407. Originally published 2025 Dec 17. [Version 2] doi: 10.12688/f1000research.173462.2 (PMC12856247; doi:10.12688/f1000research.173462.2)
Supplement: Peer review history

# Reviewer report: Reviewer response for version 2

**DOI:** 10.5256/f1000research.195345.r451929

**Published:** 2026-01-29

**Authors:** Birsel Aybek

**Affiliations:** 1 Çukurova University, Adana, Turkey

I have reviewed the revision of the article titled Mathematical Competencies and Critical Thinking in Secondary Education: A PRISMA-Based Systematic Review (2019-2025). It has been determined that the authors have provided sufficient answers to my questions

Are the rationale for, and objectives of, the Systematic Review clearly stated?

Yes

Is the statistical analysis and its interpretation appropriate?

Yes

If this is a Living Systematic Review, is the ‘living’ method appropriate and is the search schedule clearly defined and justified? (‘Living Systematic Review’ or a variation of this term should be included in the title.)

Yes

Are sufficient details of the methods and analysis provided to allow replication by others?

Yes

Are the conclusions drawn adequately supported by the results presented in the review?

Yes

Reviewer Expertise:

NA

I confirm that I have read this submission and believe that I have an appropriate level of expertise to confirm that it is of an acceptable scientific standard.

# Reviewer report: Reviewer response for version 2

**DOI:** 10.5256/f1000research.195345.r452068

**Published:** 2026-01-29

**Authors:** Geovanny Francisco Ruiz Muñoz Muñoz

**Affiliations:** 1 University of Guayaquil, Guayaquil, Ecuador

The article represents a valuable and well-founded contribution to the field of secondary education, providing a rigorous and up-to-date systematic review of the integration of mathematical competencies and critical thinking. The consistent application of the PRISMA 2020 protocol, the clarity of the research questions, and the strength of the theoretical framework lend coherence and credibility to the study. In addition, the breadth of the analysis, the methodological diversity of the included studies, and the careful synthesis of the findings offer a comprehensive overview of the state of the art, effectively highlighting the role of active, student-centered methodologies. Overall, the manuscript stands out as a relevant reference for researchers, teacher educators, and policymakers interested in fostering higher-order cognitive skills in mathematics education.

Are the rationale for, and objectives of, the Systematic Review clearly stated?

Yes

Is the statistical analysis and its interpretation appropriate?

Yes

If this is a Living Systematic Review, is the ‘living’ method appropriate and is the search schedule clearly defined and justified? (‘Living Systematic Review’ or a variation of this term should be included in the title.)

Yes

Are sufficient details of the methods and analysis provided to allow replication by others?

Yes

Are the conclusions drawn adequately supported by the results presented in the review?

Yes

Reviewer Expertise:

My areas of research include mathematics education, critical thinking development, competency-based learning, and active pedagogical methodologies in secondary education. I have particular expertise in systematic reviews and educational research methods, as well as in the analysis of student-centered approaches such as Problem-Based Learning, Project-Based Learning, STEM/STEAM education, and the use of digital technologies to support higher-order cognitive skills. This background allows me to assess both the theoretical coherence and the methodological rigor of studies examining the integration of mathematical competencies and critical thinking in school contexts.

I confirm that I have read this submission and believe that I have an appropriate level of expertise to confirm that it is of an acceptable scientific standard.

# Reviewer report: Reviewer response for version 1

**DOI:** 10.5256/f1000research.191282.r442601

**Published:** 2026-01-07

**Authors:** Birsel Aybek

**Affiliations:** 1 Çukurova University, Adana, Turkey

**The following limitations were identified in the study:**

 1.Why was the secondary school level specifically examined in the study? Why weren't elementary schools or other levels examined? Because such a problem also exists in other levels. The article needs to explain in more detail why secondary schools were chosen.

 2. Why were studies conducted specifically between 2019 and 2025 considered in this research? Is there a particular reason for choosing 2019 as the starting year? It is believed that explaining the rationale for this choice in the article would be appropriate.

 3. The interpretation of the following finding revealed in the research is important. Why are there more studies on this topic in 2023? Researchers should evaluate this.

 “Interest in integrating mathematical competencies and critical thinking skills in secondary education has shown a steady increase between 2019 and 2025. Over the last three years (2022–2024), scientific output has increased by 67%, with 2023 standing out as the most productive year, accounting for 25% of the total with six studies.”

 4. The need for such a study should be explained in greater detail in the introduction section of the article. At the same time, it remains unclear whether critical thinking influences mathematical competence or whether mathematical competence influences critical thinking. What kind of relationship exists between these two constructs? The answers to these questions should be discussed more comprehensively in the introduction section.

Are the rationale for, and objectives of, the Systematic Review clearly stated?

Yes

Is the statistical analysis and its interpretation appropriate?

Yes

If this is a Living Systematic Review, is the ‘living’ method appropriate and is the search schedule clearly defined and justified? (‘Living Systematic Review’ or a variation of this term should be included in the title.)

Yes

Are sufficient details of the methods and analysis provided to allow replication by others?

Yes

Are the conclusions drawn adequately supported by the results presented in the review?

Yes

Reviewer Expertise:

teacher education, curricula, critical thinking, and instructional methods

I confirm that I have read this submission and believe that I have an appropriate level of expertise to confirm that it is of an acceptable scientific standard, however I have significant reservations, as outlined above.

# Response

**Published:** 2026-01-15

**Authors:** Andrea Basantes-Andrade

**Affiliations:** Universidad Técnica del Norte, Ecuador

Dear Reviewer,

 We sincerely appreciate the time and effort spent evaluating our manuscript. Your comments and suggestions have been extremely valuable in strengthening the scientific quality, clarity, and overall coherence of the work.

 Below, we provide a detailed response to each comment, indicating the revisions incorporated into the manuscript.

**Comment 1.  **Why was the secondary school level specifically examined in the study? Why weren't elementary schools or other levels examined? Because such a problem also exists in other levels. The article needs to explain in more detail why secondary schools were chosen.

**Answer. **We appreciate this observation. The focus on secondary education is theoretically and pedagogically justified, as this stage is characterized by the consolidation of abstract, formal, and hypothetical-deductive reasoning, which are essential for the deep integration of mathematical competence and critical thinking. International frameworks (OECD, UNESCO) explicitly position secondary education as the key stage for developing higher-order thinking, analytical reasoning, and problem-solving skills. Therefore, the choice does not disregard other educational levels but reflects the cognitive, curricular, and evaluative density specific to secondary education.

**Text included in the second paragraph of the introduction**

 This systematic review focuses on secondary education because this stage represents a critical period for the consolidation of abstract, formal, and hypothetical-deductive reasoning, which are foundational for both mathematical competence and critical thinking development (Inhelder & Piaget, 2013; OECD, 2019). International curricular frameworks emphasize secondary education as the stage where higher-order thinking, problem solving, and analytical reasoning are explicitly articulated as learning outcomes (UNESCO, 2021; OECD, 2022). Furthermore, secondary school mathematics involves increased conceptual complexity, formalization, and transfer demands, making it a particularly relevant context to examine the integration of mathematical competence and critical thinking.

 Bibliographic reference included in the manuscript

 Inhelder, B., & Piaget, J. (2013). The growth of logical thinking from childhood to adolescence: An essay on the construction of formal operational structures. Routledge.

 Organisation for Economic Co-operation and Development (OECD): PISA 2018 results: What students know and can do. OECD Publishing; 2019. 10.1787/5f07c754-en

 United Nations Educational, Scientific and Cultural Organization: Reimagining our futures together: A new social contract for education. UNESCO; 2021. Reference Source

**Comment 2.   **Why were studies conducted specifically between 2019 and 2025 considered in this research? Is there a particular reason for choosing 2019 as the starting year? It is believed that explaining the rationale for this choice in the article would be appropriate.

**Answer. **The 2019–2025 timeframe was selected based on three criteria:

1. The recent consolidation of critical thinking as a transversal competence in international educational policies (OECD, UNESCO).
2. The expansion of active and digital methodologies following the COVID-19 pandemic. 
3. The need to capture contemporary and contextually relevant evidence on the integration of both constructs in current educational settings.

**Text included in the last paragraph of section 2.1 Information sources and search period**

 The time frame 2019–2025 was selected to capture the most recent and relevant evidence following the global curricular shift toward competency-based education and critical thinking development (OECD, 2019; UNESCO, 2021). This period also reflects the acceleration of digital transformation and active methodologies in education, particularly in response to the COVID-19 pandemic, which significantly reshaped pedagogical practices worldwide.

**Comment 3.  **The interpretation of the following finding revealed in the research is important. Why are there more studies on this topic in 2023? Researchers should evaluate this. “Interest in integrating mathematical competencies and critical thinking skills in secondary education has shown a steady increase between 2019 and 2025. Over the last three years (2022–2024), scientific output has increased by 67%, with 2023 standing out as the most productive year, accounting for 25% of the total with six studies.”

**Answer. **The concentration of studies in 2023 reflects the maturation of post-pandemic innovation projects, increased funding for educational transformation, and intensified institutional pressure to demonstrate transversal skill development. This peak aligns with global post-pandemic research trends.

**Text included in the fourth paragraph of the results section**

 The peak in publications observed in 2023 can be attributed to the maturation of post-pandemic educational innovation projects, increased institutional funding for digital transformation, and heightened policy emphasis on transversal competencies. This pattern aligns with global reports indicating a surge in research on critical thinking and competency-based education following the pandemic (UNESCO, 2022; OECD, 2023).

 Bibliographic reference included in the manuscript

 Organisation for Economic Co-operation and Development (OECD): Science, Technology and Innovation Outlook 2023: Enabling Transitions in Times of Disruption. OECD Publishing; 2023. 10.1787/0b55736e-en 

 United Nations Educational, Scientific and Cultural Organization: Transforming Education Summit. UNESCO; 2022. Reference Source.

**Comment 4.**  The need for such a study should be explained in greater detail in the introduction section of the article. At the same time, it remains unclear whether critical thinking influences mathematical competence or whether mathematical competence influences critical thinking. What kind of relationship exists between these two constructs? The answers to these questions should be discussed more comprehensively in the introduction section.

**Answer. **The introduction has been expanded to clarify that the relationship between critical thinking and mathematical competence is bidirectional, reciprocal, and dynamic, grounded in constructivist and sociocultural frameworks. It is argued that critical thinking enhances mathematical understanding, while structured mathematical activity fosters the development of critical thinking.

**Text included in the introduction section**

 The relationship between mathematical competence and critical thinking is not unidirectional but reciprocal and recursive. Mathematical reasoning provides structured contexts for analysis, argumentation, and abstraction, while critical thinking enhances conceptual understanding, problem interpretation, and metacognitive regulation in mathematics (OECD, 2019; Pratiwi et al., 2025; Pereira-González et al., 2024; Rahmatika et al., 2024). Recent empirical studies confirm that students who engage in mathematical tasks requiring justification, modeling, and decision-making simultaneously develop higher levels of critical evaluation, cognitive flexibility, and reflective thinking (Li & Oon, 2024; Er, 2024; Pramasdyahsari et al., 2023). This interdependence positions both constructs as mutually reinforcing rather than hierarchically ordered, suggesting that their integration is pedagogically synergistic rather than sequential.

 We sincerely thank you for your time, consideration, and the opportunity to submit our manuscript for evaluation. We remain at your disposal for any additional information or clarification you may require.

 Kind regards,

 Andrea Basantes-Andrade
